# Kawaii-Ness Mediates Between Demographic Variables, Happiness, and Brain Conditions

**DOI:** 10.3390/brainsci15030289

**Published:** 2025-03-09

**Authors:** Keisuke Kokubun, Kiyotaka Nemoto, Taiko Otsuka, Maya Okamoto, Yuko Shiga, Yuya Makizato, Aya Komaki, Yoshinori Yamakawa

**Affiliations:** 1 Graduate School of Management, Kyoto University, Kyoto 606-8501, Japan; 2 Department of Psychiatry, Institute of Medicine, University of Tsukuba, Tsukuba 305-8577, Ibaraki, Japan; 3 Sanrio Entertainment, Co., Ltd., Tama 206-8588, Tokyo, Japan; 4 Institute of Innovative Research, Tokyo Institute of Technology, Meguro 152-8550, Tokyo, Japan; 5 ImPACT Program of Council for Science, Technology and Innovation, Cabinet Office, Government of Japan, Chiyoda 100-8914, Tokyo, Japan; 6 Office for Academic and Industrial Innovation, Kobe University, Kobe 657-8501, Hyogo, Japan; 7 Brain Impact, Kyoto 606-8501, Japan

**Keywords:** demographic scales, fractional anisotropy, happiness, kawaii-ness

## Abstract

**Background/Objectives**: In many societies, especially in highly masculine societies like Japan, being a man, getting older, engaging in knowledge work, and earning a high annual salary are seen as conditions for success. On the other hand, an increasing number of studies have shown that incorporating kawaii-ness into our lives can help maintain and improve happiness and well-being. **Methods**: Therefore, in this study, we employed a variable expressing the response to kawaii-ness together with four demographic variables (sex, age, income, and knowledge work), happiness, and fractional anisotropy brain healthcare quotient (FA-BHQ) which is derived from magnetic resonance imaging (MRI) images calculations to analyze the relationship between them. **Results**: The results of a path analysis using data obtained from 182 healthy men and women showed that kawaii-ness mediates the association between demographic variables and happiness, which is in turn associated with FA-BHQ. Furthermore, with the correlation analysis between happiness and individual FA regions, we were able to confirm that FA regions, including the limbic-thalamo-cortical pathway, which is responsible for emotional regulation, are related to happiness. **Conclusions**: These results indicate the following: Men, older people, people engaged in knowledge work, and people with high annual incomes avoid kawaii-ness; As a result, they are unable to obtain the sense of happiness that they should have; as a result, they are unable to keep their brains healthy, and their brain functions, including emotional regulation, are not functioning properly; This may prevent them from maintaining or improving their performance. This study is the first attempt to clarify the relationship between demographic scales, kawaii-ness, happiness, and brain conditions.

## 1. Introduction

In many societies, especially in highly masculine societies like Japan, being a man, getting older, engaging in knowledge work, and earning a high annual salary are seen as conditions for success [1,2]. In addition to these demographic variables, kawaii-ness, which is increasingly getting popular around the world [3,4], can be considered a determining factor of happiness and well-being. Kawaii, a Japanese adjective that means cute, beautiful, nice, adorable, and so on, is used in daily situations to express a favorable evaluation of an object or a person [5]. Kawaii-ness influences people’s behavior by inducing positive emotional states [6,7]. For example, a character with physical characteristics such as a large head, large eyes, high forehead, chubby cheeks, small nose and mouth, short and thick limbs, and a plump figure [8], which are perceived as cute, has gained tremendous popularity because of human emotional response to kawaii-ness [4]. Previous studies have shown that products with kawaii-ness as an element can heal users [9,10] and reduce stress [11,12], and have been shown to increase people’s sense of well-being. Feeling kawaii also triggers activity in the zygomaticus muscle, which is used for smiling [13], and promotes social relationships [14]. Simultaneously, it was reported that engaging in kawaii-ness increases empathy [14] and attention [6]. Furthermore, some research has shown that increasing one’s kawaii-ness increases one’s chances of being accepted in an unfamiliar community [14]. These findings suggest that incorporating kawaii-ness into life can improve social skills and make it easier for individuals to feel happy.

However, Japan is the birthplace of kawaii-ness, but at the same time, it is a society characterized by masculinity. While focusing on social success such as making money, they do not value non-monetary things very favorably. This tendency is very different from Western societies, which emphasize humanity and freedom [2]. This may lead not only to the low level of non-economic activities such as charity and volunteer work in Japan [15], but also to low happiness [16], high ethnocentrism [17], and a high number of dementia patients [18]. In other words, low interest in things other than work may lead to them losing opportunities for diverse enjoyment and interaction, discriminating against people from different cultures who value life outside of work, and finding no alternative to work after retirement, which could accelerate brain aging. Recently, we have shown that the gray matter of the triple brain network, which controls judgment, empathy, and the switching between the two, may be related to understanding diversity, including gender and origins [19]. Furthermore, in the field of neuroaesthetics, it has been shown that neural responses to art may activate reward circuits [20,21]. Thus, engagement with diversity and art may improve brain health. Similarly, research on the effects of kawaii-ness is accumulating. For example, in the field of consumer psychology, it has been shown that products with kawaii-ness increase consumers’ willingness to purchase them [22,23]. In the field of emotion regulation theory, it has been shown that viewing kawaii images may promote cautious behavior and narrow the focus of attention [7]. In the field of positive psychology, it has been shown that kawaii-ness may promote prosocial behavior [24] and mutual help [25]. These suggest that people who avoid kawaii-ness may not be able to take appropriate actions that they would have been able to take, and therefore may miss opportunities to improve happiness and brain health that they could have taken. In other words, it is suggested that the degree of response to kawaii-ness may mediate the relationship between demographic information, happiness and brain health.

Therefore, in this study, we aim to demonstrate that kawaii-ness is a determining factor of happiness, mediating the relationship between demographic variables and happiness. To achieve this, we incorporate a psychological scale that reflects responsiveness to kawaii-ness into an analytical model. Furthermore, within the same analytical model, we confirmed that happiness obtained through high kawaii-ness is correlated with fractional anisotropy brain healthcare quotient (FA-BHQ), an index obtained through brain image analysis. Fractional anisotropy (FA) is widely used in neuroscience research as a measure reflecting the microstructural integrity of white matter [26] and has been used to assess the progression of pathologies such as Parkinson’s disease [27] as well as emotion regulation [28] and cognitive ability [29]. Furthermore, in recent years, a correlation between FA and happiness has been confirmed [30,31]. Happiness here refers to “the experience of joy, contentment, or positive well-being, combined with a sense that one’s life is good, meaningful, and worthwhile” ([32]: p. 32) which includes the meanings of both hedonic (pleasure-seeking) and eudaimonic (meaning-driven) happiness. Therefore, by demonstrating the relationship between happiness and FA-BHQ in this study, it is possible that the happiness obtained by incorporating kawaii-ness into daily life can lead to better performance in social life through the maintenance and improvement of brain conditions. This study was the first attempt to clarify the relationship between demographic scales, kawaii-ness, happiness, and brain conditions.

The “baby schema”, which humans perceive as “cute”, is a concept proposed by ethologist Konrad Lorenz and refers to characteristics such as “a large head relative to the size of the body”, “large round eyes at the bottom of the face”, and “a rounded body type”, which increase the individual’s chances of survival by eliciting nurturing behavior from observers [33]. Kawaii, a Japanese adjective meaning cute, good, pretty, adorable, and lovely, is used in everyday situations to express a favorable evaluation of an object or person and refers to the emotion that is generated in the viewer [5], while cuteness describes an attribute of the object. Furthermore, kawaii-ness not only induces protection in the viewer, but also the desire to continue looking at the object and coexist with it [5], and is also associated with playfulness [34]. Thus, kawaii-ness is a multifaceted construct with several fundamental aspects, while having a unified general concept at its core [34]. Therefore, although scales for measuring reactions to kawaii-ness have been developed in several previous studies [35], in our study, we use the Kawaii Reactivity Index (KRI) developed by Umesawa et al. [36] and details have been presented in our previous study [37]. Through this approach, we aim to clarify the relationship between happiness and reactions to various types of kawaii-ness incorporated into daily life, and not just reactions to human and animal babies. Additionally, we used the Subjective Happiness Scale (SHS) [38], a representative scale for measuring happiness, along with gray matter brain healthcare quotient (GM-BHQ) and FA-BHQ, as indicators of happiness and brain conditions, respectively.

## 2. Materials and Methods

### 2.1. Participants

To the best of our knowledge, no cross-sectional study has shown a direct correlation between kawaii-ness or cuteness and happiness or well-being. Therefore, considering that kawaii-ness is a strongly felt reaction towards human and animal babies [36], the sample size was determined based on the correlation coefficients between SHS and the feelings that parents have toward their children, such as “joys of child-care” (r = 0.27, *p* < 0.001) and “connection with the child” (r = 0.16, *p* < 0.001), measured with a short form of Childcare Happiness Scale [39]. The required sample size of “175” was calculated using G*Power 3.1.9.7, assuming an effect size of 0.21 determined by the average value of these correlation coefficients, an α error probability of 0.05, and power (1–β error prob) of 0.8. We aimed to recruit 185 participants, with 10 participants in reserve.

**Figure 1:**
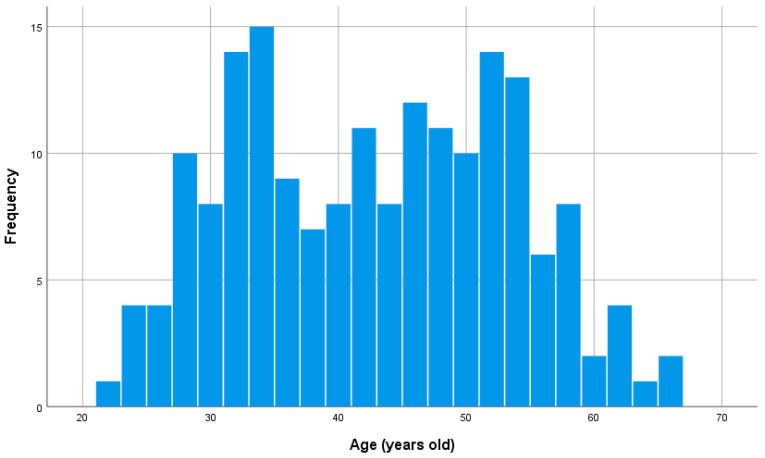
Distribution of age.

Between January 18 and June 17, 2022, 186 healthy adults aged 22–66 years (97 females and 89 males) were recruited from Tokyo. Of these, 91 were recruited from a toy manufacturer’s fan community (Group 1) and 95 from users of a smartphone app for businesspeople developed by an IT company (Group 2). The former prevents the difficulty of answering questions from affecting the results by including only people who are indifferent to kawaii-ness in the sample. The latter group was selected because it is likely familiar with the latest digital devices and accepts various forms of kawaii-ness in the future. In this study, 182 people aged 22–66 years (96 women, 86 men; 90 Group 1, 92 Group 2) were used, excluding four people whose brain image information was unobtained. According to the self-reports, none of the recruited participants had records of neurological, psychiatric, or other medical conditions that could affect the central nervous system. This study was approved by the Ethics Committee of the Tokyo Institute of Technology (Approval Numbers 2021173 and 2022130), and all methods were carried out according to the relevant guidelines, regulations, and principles of the Declaration of Helsinki. All participants provided written informed consent before participating, and their anonymity was maintained. Figure 1 shows a histogram of age distribution.

### 2.2. MRI Data Acquisition

All magnetic resonance imaging (MRI) data were collected using a 3 Tesla MRI scanner (MAGNETOM Prisma, Siemens, Munich, Germany) with a 32-channel head array coil. High-resolution structural images were acquired using a three-dimensional (3D) T1-weighted magnetization-prepared rapid-acquisition gradient-echo pulse sequence. The parameters were as follows: repetition time (TR), 1900 ms; echo time (TE), 2.52 ms; inversion time (TI), 900 ms; flip angle, 9°; matrix size, 256 × 256; field of view (FOV), 256 mm; and slice thickness, 1 mm. Diffusion tensor imaging (DTI) data were collected using spin-echo echo-planar imaging (SE-EPI) with generalized auto-calibrating partially parallel acquisitions (GRAPPA). Image slices were parallel to the orbitomeatal (OM) line. The parameters were as follows: TR = 14,100 ms; TE = 81 ms, flip angle = 90°, matrix size = 114 × 114; FOV  =  224 mm, and slice thickness = 2 mm. Baseline images (b = 0 s/mm^2^) and 30 different diffusion orientations were acquired with a b value of 1000 s/mm^2^.

### 2.3. MRI Data Analysis

T1-weighted images were preprocessed and analyzed using Statistical Parametric Mapping 12 (SPM12; Wellcome Trust Center for Neuroimaging, London, UK) in MATLAB R2020b (MathWorks Inc., Sherborn, MA, USA). Each MPRAGE image was split into gray matter (GM), white matter (WM), and cerebrospinal fluid (CSF) using the SPM12 prior probability template. Subsequently, diffeomorphic anatomical registration using the exponentiated lie algebra (DARTEL) algorithm [40] was used to spatially normalize the segmented GM images. A modulation step was also incorporated into the preprocessing model to reflect the regional volume and preserve the total GM volume before warping. As a final preprocessing step, we smoothed all segmented, modulated, and normalized images using an 8 mm full width at half-maximum (FWHM) Gaussian kernel. The intracranial volume (ICV) was calculated by adding the GM, WM, and CSF images for each participant. To control for differences in whole-brain volume between participants, proportional GM images were generated by dividing the smoothed GM image by the ICV. The mean and standard deviation (SD) images were generated for all participants using proportional GM images. We then calculated the GM-BHQ and defined the mean as BHQ 100 and the SD as 15 BHQ points. According to this definition, approximately 95% of the population scored between BHQ70 and BHQ130. Individual GM quotient images were calculated using the following formula: 100 + 15 × (individual proportional GM − mean)/SD. Subsequently, using an automated anatomical labeling (AAL) atlas [41], regional GM quotients were extracted and averaged across regions to create a participant-specific GM-BHQ.

The DTI data were preprocessed using the FMRIB software library (FSL) 6.0.2 [42]. First, in the initial b0 image, all diffuse images were aligned, and motion and distortion corrections were performed using eddy correction. FA images were computed using DTIFit according to these corrections. The FA images were then spatially normalized to the standard Montreal Neurological Institute (MNI) space using FLIRT and FNIRT. Mean and standard deviation (SD) images were generated from all FA images after spatial normalization. Individual FA quotient images were then calculated using the following formula: 100 + 15 × (individual FA − mean)/SD. Finally, using the Johns Hopkins University (JHU) DTI-based white matter atlas [43], regional FA quotients were extracted and averaged across the regions to yield a participant-specific FA-BHQ. For more details, refer to our previous research [44,45,46].

The reason for using the GM-BHQ and FA-BHQ is that these indices have been used primarily in studies of healthy middle-aged adults, as in this study, rather than in elderly people or people with underlying diseases, and correlations with various psychological indices have been confirmed. In our previous studies, whole-brain GM-BHQ was positively correlated with dietary balance [47] and negatively with unhealthy lifestyle [48], while the whole-brain FA-BHQ was positively correlated with cognitive function [29] and happiness [30] and negatively with anxiety [49]. In addition to the entire brain, we defined eight subregions of the FA-BHQ as regions of interest (ROI). Previous studies have shown that FA regulates emotions in some regions of the limbic-thalamic-cortical pathway such as the corona radiata, internal capsule, and cingulum [28,50,51,52]. In contrast, others have discussed that the superior longitudinal fasciculus [53,54,55,56], corpus callosum [57,58,59], or prefrontal cortex/uncinate fasciculus/amygdala pathway [60,61,62,63] play an even more important role in processing emotions. Our study, which focuses on brain GMV and FA, differs from studies using other neural biomarkers such as functional MRI (fMRI) and cortical thickness. fMRI is good at examining brain responses, but it cannot tell us the lasting effects of brain influences [64]. Cortical thickness may be better than GMV at observing genetic effects, but GMV, which is a function of surface area and cortical thickness, is more comprehensive [65]. In addition, FA is an index that reflects the uniform orientation of white matter fibers and is highly sensitive to changes in brain microstructure [66]. This study aims to clarify the lasting, comprehensive, and detailed effects of adopting kawaii-ness by using GMV and FA.

### 2.4. Psychological Test

#### 2.4.1. A Scale for Measuring Happiness

**Figure 2:**
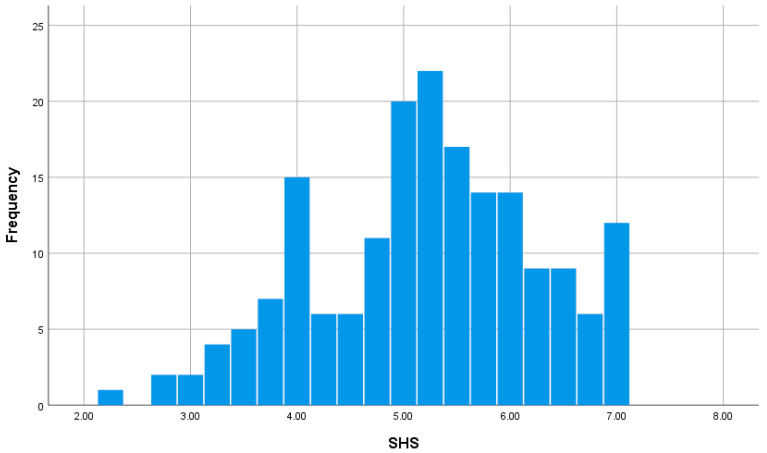
Distribution of SHS scores.

We used the SHS, which was developed to assess subjective happiness and consists of four items assessed on a 7-point Likert scale [38], to measure happiness. For the first two items, respondents were required to rate their general happiness and happiness compared to their peers (1 = less happy to 7 = very happy). The other two items provide a brief description of generally happy and unhappy people. Respondents were asked to indicate the extent to which each trait describes them (1 = not at all to 7 = a great deal). The arithmetic mean values of the four items were used in the analysis. The reliability coefficient (Cronbach’s α) of the SHS used in this study was 0.835, which is sufficiently high. The current study used SHS, as this scale is one of the most widely used well-being measures internationally [67]. Figure 2 shows a histogram of the distribution of the SHS scores.

#### 2.4.2. A Scale for Measuring Kawaii-Ness and Its Validation

**Figure 3:**
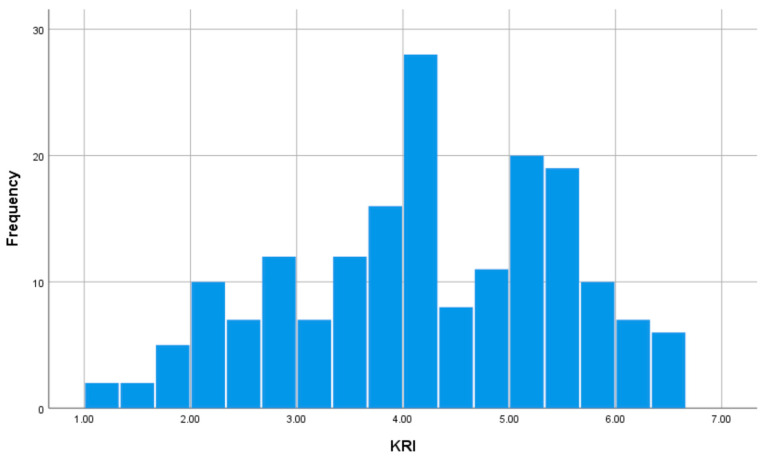
Distribution of KRI scores.

We used the total score of the 36-item KRI developed by Umesawa et al. [36] to capture kawaii-ness from a multidimensional perspective. Respondents were asked to respond to the items on a 7-point scale, ranging from one (not at all) to seven (strongly). The scree plot obtained by varimax rotation principal component analysis using responses to the 36 KRI items had eigenvalues of 15.853, 2.727, 1.888, 1.557, 1.141, 1.071, and 0.912 for the first to the seventh principal components, respectively, showing that there was a large drop between the first and second components. The contribution of the first component was 44.036%. Furthermore, the commonality ranged from 0.508 to 0.826, with no items showing low values. Thus, it is appropriate to consider KRI as a one-factor structure. The Cronbach’s α of the KRI used in this study was 0.962, which is sufficiently high. Figure 3 shows a histogram of the distribution of the KRI scores.

Additionally, to verify that the KRI was the scale intended by the developer, we employed a uniquely developed single scale, the 12-item Kawaii Likeability and Adoption Scale (KLAS), to measure the degree of liking and adopting of six characters related to kawaii-ness. We also used the 13-item Behavioral Approach system (BAS) developed by Carver and White [68], which is used to measure reward reactivity, because previous research has shown that people with high BAS respond more to cuteness [69] and are more proactive in nurturing and caring toward their children [70] than those with low BAS. Like the KRI, the scree plot obtained by principal component analysis with varimax rotation using the responses to the 12 items of the KLAS indicated that the eigenvalues varied from 7.554 to 1.184, 0.860, and 0.622, among others, showing that there was a large drop between the first and second factors. The contribution of the first component was 62.949%. Furthermore, the commonality ranged from 0.577 to 0.828, with no items showing low values. These results indicate that it is appropriate to consider the KLAS as a one-factor structure. Cronbach’s α for KLAS and BAS in this research were 0.946 and 0.877, respectively, which was sufficiently high. The mean value (M) and standard deviation (SD) were KLAS (M = 3.291; SD = 1.567) and BAS (M = 3.218; SD = 0.496), respectively. Considering that KRI is an index that measures the “reactivity” that occurs when experiencing “kawaii-ness” [36], it was expected that KRI would show a positive and significant correlation with KLAS and BAS. The correlation coefficient between KRI and KLAS (r = 0.715, *p* < 0.001), KRI and BAS (r = 0.458, *p* < 0.001), and KLAS and BAS (r = 0.294, *p* < 0.001) were consistent with the expectation. This implies that the KRI is a valid scale for measuring responsiveness to kawaii-ness.

### 2.5. Demographic Scales

Knowledge work, annual income, age, sex, and body mass index (BMI, kg/m^2^) were adopted as demographic scales. Knowledge work was a binary variable that takes the value of 1 when the occupations “managerial” or “professional/technical” are selected, and 0 when neither is selected from the following 13 occupations: “managerial”, “professional/technical”, “office work”, “sales”, “service”, “security”, “agriculture, forestry, or fishery”, “production process”, “transportation/mechanical operation”, “construction/mining”, “carrying/cleaning/packaging”, “student”, and “unemployed”. Although many different views exist on what constitutes a knowledge worker, we follow Drucker [71] who defined knowledge workers as ‘professional, managerial, and technical people’. The annual income was created by allocating 1–16 points for each of the following 16 options: “No income”, “<500,000 yen”, “500,000–990,000 yen”, “1–1.49 million yen”, “1.5–1.99 million yen”, “2–2.49 million yen”, “2.5–2.99 million yen”, “3–3.99 million yen”, “4–4.99 million yen”, “5–5.99 million yen”, “6–6.99 million yen”, “7–7.99 million yen”, “8–8.99 million yen”, “9–9.99 million yen”, “10–14.99 million yen”, and “≥15 million yen”. The actual age answered by the respondent was used in the analysis. In addition, sex was converted into a binary variable with a value of 1 for males and 0 for females. Although unrelated to the hypothesis, BMI (kg/m^2^) which is said to be an index that holistically reflects a person’s health status [72] was calculated using height and weight to control the health status of participants.

### 2.6. Data Analysis

First, the Student’s *t*-test and chi-square test were performed to confirm the difference in the values of variables between different groups. Then, correlation analysis between variables was performed using a pooled data set of values for both groups. The results of the Kolmogorov–Smirnov normality test showed that SHS and KRI did not follow the normal distribution, so Spearman’s rho value was shown. However, the residuals of the regression with demographic variables (Knowledge work, Income, Age, Sex) as independent variables followed normal distribution for SHS, KRI, FA-BHQ, and GM-BHQ, so Pearson’s partial correlation coefficients controlled for these variables were also shown. Next, path analysis was performed using the maximum likelihood method with Amos software (version 26.0; IBM Corporation Software Group, Somers, NY, USA) to test the relationship between variables. We performed a mediation analysis using maximum likelihood bootstrapping to test whether KRI mediated between demographic variables and SHS. Maximum likelihood bootstrapping is recommended for analyzing data showing non-normal distributions, such as in this study [73]. Finally, we performed a Pearson partial correlation analysis between FA regions and SHS. Additionally, SPSS 26.0 software (IBM Corporation Software Group, Armonk, NY, USA) was used for all the other analyses. The significance level for all analyses was two-sided 5%. The model fit of path analysis was examined using fit indices: root mean square error of approximation (RMSEA); goodness of fit index (GFI), adjusted goodness of fit index (AGFI), normed fit index (NFI), and comparative fit index (CFI). GFI, AGFI, NFI, CFI and IFI indices should be above 0.9, PCLOSE should be above 0.5, whereas RMSEA has to be less than 0.05 [74].

## 3. Results

Table 1 presents descriptive statistics. There was a significant difference in KRI (t = 10.259, *p* < 0.001), age (t = 3.558, *p* < 0.001), income (t = 10.366, *p* < 0.001), GM-BHQ (t = 3.599, *p* < 0.001), sex (χ^2^ = 30.272, *p* < 0.001), and knowledge work (χ^2^ = 36.930, *p* < 0.001). In other words, compared with Group 2, Group 1 tended to be more accustomed to kawaii-ness, were younger, had a larger GM-BHQ, and had a lower income level. In addition, Group 1 had more women and more people engaged in non-knowledge work than Group 2 did. Table 2 shows the correlations between the variables. The figures below the diagonal are Spearman’s ρ. KRI was significantly positively correlated with SHS (r = 0.184, *p* = 0.013) and GM-BHQ (r = 0.324, *p* < 0.001), and negatively correlated with Knowledge work (r = −0.411, *p* < 0.001), Income (r = −0.497, *p* < 0.001), Age (r = −0.252, *p* < 0.001), and Sex (r = −0.530, *p* < 0.001). The figures above the diagonal are Pearson’s correlation coefficients controlling for demographic variables (knowledge work, income, age, sex). SHS was significantly positively correlated with KRI (r = 0.251, *p* = 0.001) and FA-BHQ (r = 0.228, *p* = 0.002). GM-BHQ was significantly negatively correlated with BMI (r = −0.300, *p* < 0.001).

**Figure 4:**
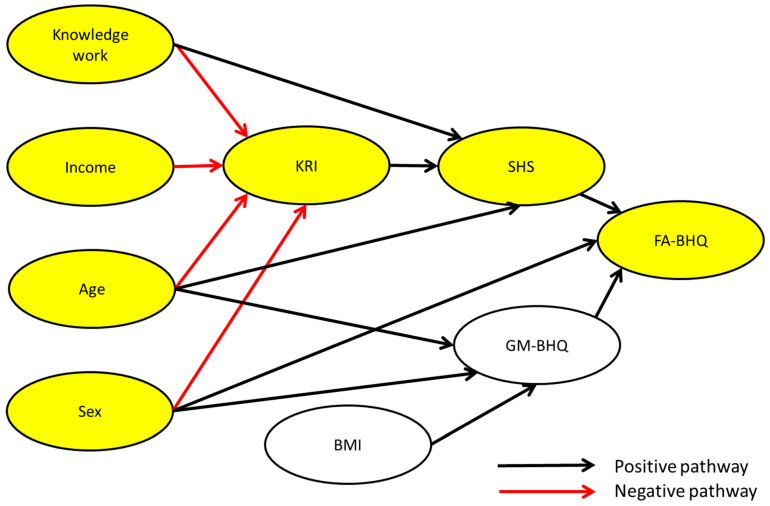
Path diagram for the resulting association between knowledge work, age, sex, income, KRI, BMI, SHS, the GM-BHQ, and the FA-BHQ. All the paths are significant at the 5% level, except for the path from knowledge work to cuteness, which is 0.051. Goodness-of-fit indices: χ^2^ = 24.532; df = 18; root mean square error of approximation (RMSEA) = 0.045; probability of close fit (PCLOSE) = 0.539; goodness of fit index (GFI) = 0.971; adjusted goodness of fit index (AGFI) = 0.926; normed fit index (NFI) = 0.947; comparative fit index (CFI) = 0.985. n = 182. Error terms and correlations between variables are omitted in the figure. The yellow variables are the hypothesized variables. BMI: body mass index (BMI, kg/m^2^).

As shown in Table 3 and Figure 4, path analysis, which cleared the thresholds of fit indices, showed that knowledge work (β = −0.129, *p* = 0.051), income (β = −0.308, *p* < 0.001), and age (β = −0.139, *p* < 0.05) had negative correlations with KRI. In turn, KRI (β = 0.318, *p* < 0.001) had a positive correlation with SHS. Therefore, KRI mediates the relationship between these demographic variables and SHS, indicating that people who are engaged in knowledge work, who have a high income, and who are older tend to keep kawaii-ness away and that they are not experiencing enough happiness and they should be experiencing more. Likewise, there is a negative correlation between sex (β = −0.322, *p* < 0.001) and KRI, indicating that males are less likely to incorporate kawaii-ness into their lives than females. Furthermore, SHS (β = 0.218, *p* < 0.01) was positively correlated with FA-BHQ, indicating that high happiness leads to high FA. As a rule, path diagrams only displayed paths that were significant at the 5% level; however, a path from knowledge work to KRI which was marginally insignificant (*p* = 0.051) was retained to aid the interpretation of the results confirming that there is no change in the overall model fit.

Considering that this study was conducted on two different groups of employees, a simultaneous multi-population analysis was performed to test invariance in the magnitude of the path coefficients between the groups. There was a significant difference at the 0.1% level for the covariance of income and knowledge work and the covariance of sex and income between the groups (details available upon request). However, no other paths or covariances were significantly different at the 5% level between the groups. This demonstrates the robustness of the proposed model.

We evaluated the mediating role of KRI on the relationship between SHS and the four demographic variables (knowledge work, age, sex, and income) by the mediation tests using 5000 bootstrap samples, maximum likelihood estimators, and 95% bias-corrected confidence intervals. The results showed that: the direct effect of knowledge work (β = 0.192, *p* = 0.030) and age (β = 0.186, *p* =0.011) on SHS was positive and significant while the direct effect of income (β = 0.131, *p* = 0.109) and sex (β = −0.157, *p* = 0.074) were insignificant; indirect effect of knowledge work (β = −0.040, *p* = 0.031), income (β = −0.096, *p* = 0.000), sex (β = −0.100, *p* = 0.000), and age (β = −0.043, *p* = 0.012) were all minus and significant. Thus, KRI was shown to partially mediate the relationship between knowledge work, age and SHS and fully mediate the relationship between income, sex, and SHS. An overview of the mediation analysis is presented in Table 4.

Table 5 shows the correlation between the FA-BHQ scores for each region and the SHS. The model was controlled for sex and the GM-BHQ, which were correlated with the FA-BHQ in the path analysis. Since both the controlled FA-BHQ and SHS followed normal distributions, Pearson’s partial correlation coefficients are shown here. Like Kokubun et al. [30], we used seven regions that are related to emotion regulation that were identified in previous studies. The analysis showed that each region had a significant correlation with the SHS at the 5% level. In addition, multiple comparisons using the Benjamani–Hochberg method showed a significant correlation with the SHS at the 5% level in all regions.

**Figure 5:**
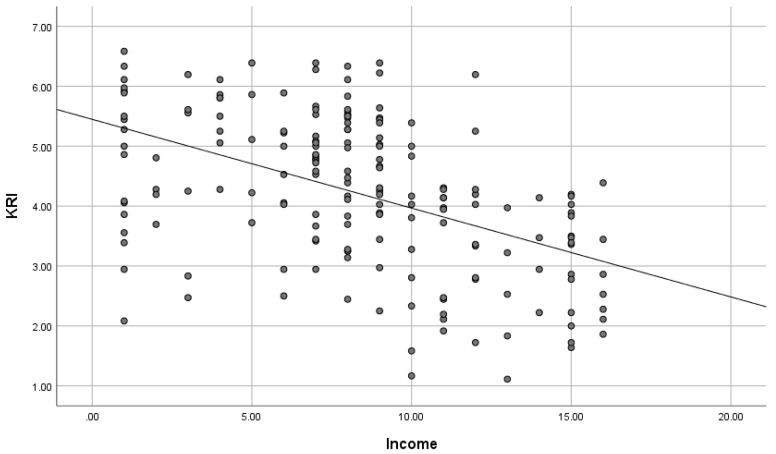
Scatter plot showing the relationship between income and KRI. The straight line in the figure is a regression line.

**Figure 6:**
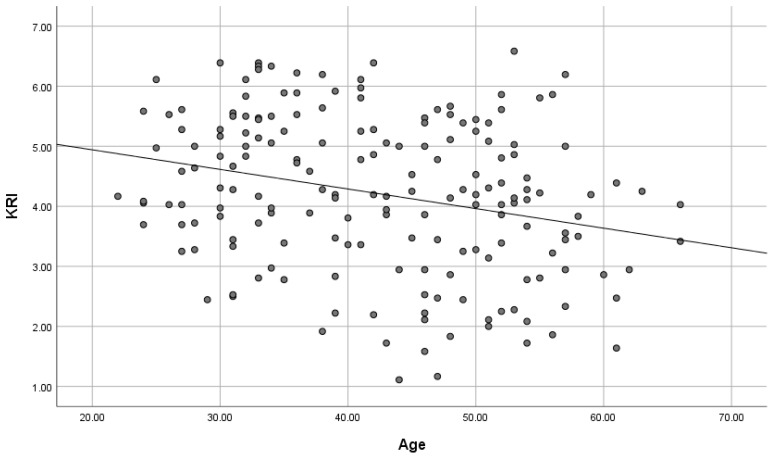
Scatter plot showing the relationship between age and KRI. The straight line in the figure is a regression line.

**Figure 7:**
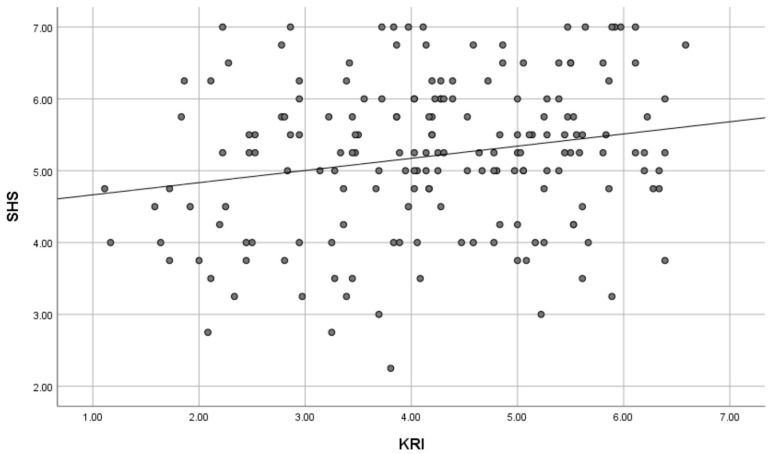
Scatter plot showing the relationship between KRI and SHS. The straight line in the figure is a regression line.

**Figure 8:**
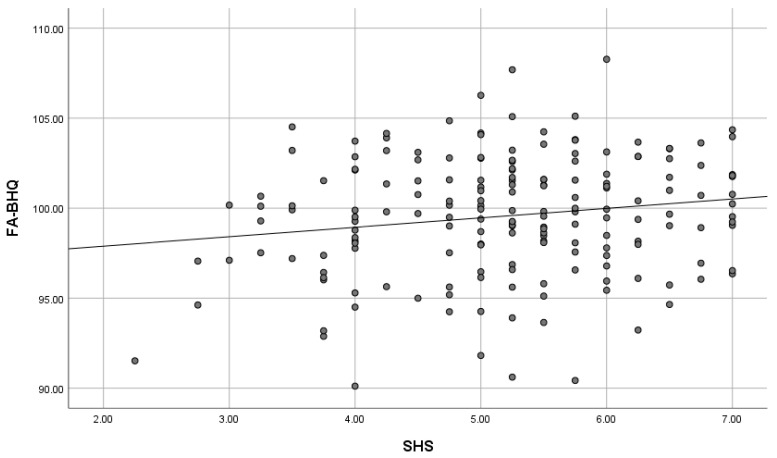
Scatter plot showing the relationship between SHS and FA-BHQ. The straight line in the figure is a regression line.

Figure 5, Figure 6, Figure 7 and Figure 8 are scatter diagrams showing the relationship between income and KRI, age and KRI, KRI and SHS, and SHS and FA-BHQ, respectively.

## 4. Discussion

In many societies, especially in highly masculine societies like Japan, being a man, getting older, engaging in knowledge work, and earning a high annual salary are seen as conditions for success [1,2]. Japanese people’s lack of interest in anything other than work could lead to a loss of diverse opportunities for enjoyment and social interaction, discrimination against people from different cultures who place importance on life outside of work, and even a lack of options outside of work after retirement, which could accelerate brain aging [15,16,17,18]. Meanwhile, an increasing number of studies have shown that kawaii-ness leads to well-being by promoting healing and stress reduction [9,10,11,12]. Furthermore, happiness is related to the brain conditions [30]. This study aimed to clarify the relationship between demographic variables, kawaii-ness, happiness, and in turn, brain conditions. As a result, it was found that the degree of kawaii-ness incorporated into life mediates the relationship between demographic variables, including knowledge work (managerial/professional), annual income, age, sex, and happiness. Furthermore, these demographic variables had negative correlations with kawaii-ness, suggesting that a certain group of people, typically older men who work in knowledge jobs and have higher annual incomes are likely to avoid kawaii-ness, resulting in lower happiness.

Previous studies have shown that there is no difference between men and women in reaction speed when they see kawaii things [75,76]. However, another previous study showed that women were more motivated than men to care for their children [77]. These differences in male and female behavior have been attributed in part to hormonal levels that are evolutionarily shaped [78] and in part to social construction [79]. In other words, men may refuse to accept kawaii-ness as it can be considered non-masculine. This is also true of knowledge workers, high-income earners, and seniors because in a society like Japan, which is extremely patriarchal and where social morals are predominantly created from a male perspective, abandoning feminine traits, and conforming to masculine traits is considered the key to social success. Hofstede revealed that, based on the results of questionnaire surveys conducted in more than 50 countries around the world, Japan is the most “masculine” country. In other words, Japan has a culture that emphasizes earning money and being promoted but does not emphasize improving the quality of life [2]. Therefore, some of the results of this study may have been influenced by the national characteristics of the Japanese people.

Furthermore, the path model used in the analysis shows that happiness, which increases with kawaii-ness, correlates with whole-brain FA-BHQ calculated by MRI image analysis, consistent with previous studies that have shown that happiness, well-being, and life satisfaction [30,80,81] are related to WM-related indicators including FA. In addition, multiple regions of FA-BHQ, including the limbic-thalamo-cortical pathway, which is said to be responsible for emotion regulation, were correlated with SHS, consistent with our previous research [30]. These findings suggest that maintaining and improving happiness may maintain and change brain structure in a way that maintains and improves emotion regulation functions.

The results of this study provide suggestions for designing new social systems. Consistent with the relationship between happiness and the brain seen above, review articles [82,83] indicate that happiness is a predictor of professional career success. Compared to unhappy people, happy people are more likely to have fewer relationship problems [84], stay in their jobs for longer [85], find another job more quickly if they lose their job [86], and exhibit higher performance [87]. Furthermore, a recent study has shown that happiness promotes altruistic behaviors [88]. Therefore, spreading kawaii-ness to a demographic that has traditionally been passive about it (e.g., male, senior, knowledge worker, etc.) may make more people happy and their brains healthier.

The results of this study provide important insights into future work styles, education, and mental health, greatly enhancing the potential of kawaii-ness. This is because the pursuit of happiness in the workplace is a goal for many managers who aim to develop their organizations. In particular, in traditional workplaces with a large number of male and older people, placing kawaii design elements in the work space is expected to increase their happiness and have a positive effect on the brain, thereby improving work performance. Furthermore, such changes may attract women and young people who want to distance themselves from masculine culture, improving the diversity of human resources. In addition, integrating kawaii-themed teaching materials in the field of education may increase students’ motivation for learning and social engagement, while at the same time encouraging the creation of values that are different from those typical of masculine culture, which places financial success as the standard of success. In this way, more people may be able to increase their happiness and improve their brain health, as well as enrich their lives after retirement, which may be effective in improving social issues such as dementia.

We would also like to address some critical arguments against kawaii-ness. Kawaii-ness has been popular in Japan since the 1970s, and one theory is that its origins date back to Japan’s defeat in World War II. Linguist Kumiko Sato [89] concluded that after the defeat in World War II and the postwar reconstruction period, Japan’s culture was prone to dependency and indulgence, and that political and economic dependence on the United States was the key to its success. This dependency fostered Japan’s “childish” popular culture and shifted from the desire to protect (cute) to the desire to be protected (kawaii) [89]. Relatedly, one theory is that the growing popularity of kawaii-ness in Japan, and more recently in China, reflects the desire for self-liberation among young people [90]. Compared to Western societies, which emphasize humanity and freedom, repression and regulation are more prominent in Eastern societies [2]. The latter’s depressing atmosphere is further intensified by the economic stagnation and slowdown of these countries [90]. In this way, kawaii-ness is, in a sense, a mirror that reflects the negative aspects of Japanese people, and for that reason, it may be easy to separate young people and women who like it from older people and men who want to avoid it. Therefore, this study, which revealed the positive psychological and neuroscientific aspects of kawaii-ness, provides a rationale for overcoming such conflicts and popularizing kawaii-ness to realists who do not want to depend on indulgence.

This is the first study to show that the introduction of kawaii-ness can increase happiness and improve brain health. In the future, we hope to further investigate and explore the effects of kawaii-ness on the brain using a variety of neuroimaging techniques, which will broaden the direction of future research and contribute to improving people’s well-being.

## 5. Limitations

This study has limitations. First, the study targeted healthy adults and did not target adolescents or elderly groups who may perceive kawaii elements differently (e.g., acceptance of anime culture, elderly care needs, etc.), so the results of this study may not be directly applicable to other age groups. Second, the sample was from Tokyo, the capital of Japan, which has the highest masculinity index in the world and a higher collectivism index than Europe and the United States on Hofstede’s dimensions, so caution should be exercised when generalizing the conclusions to other cultural systems. Third, the self-designed KRI scale did not undergo a test–retest process, which leaves concerns about the reproducibility of the results. Fourth, this study relied only on the SHS to evaluate happiness. Deeper insights into happiness may have been gained by expanding the scope of happiness to the PERMA model (positive emotions, engagement, relationships, meaning, and accomplishment) and psychological well-being, emotional stability, and life satisfaction scales. Fifth, although kawaii-ness was identified as a mediator in this study, other potential factors that affect happiness include social support, stress management, and personality traits. By incorporating these variables into the model, we may have been able to improve the accuracy of the analysis. Sixth, although this study showed that men are less likely to be involved in kawaii culture, which may affect their happiness and brain health, in addition to sex, gender norms and social expectations may actually be involved as moderating factors. Alternatively, older people with strong traditional values may avoid kawaii culture due to their social roles (work stress, family responsibilities), so these effects may have been controlled for using stress indicators (e.g., perceived stress scales). Relatedly, a design that clarifies generational differences may have allowed for deeper analysis. For example, we may have been able to compare the acceptance of kawaii culture and its impact on happiness in different generational groups (e.g., Generation Z and Generation X) and determine whether the impact is age-related or culturally influenced. Seventh, by measuring brain responses using fMRI or other methods, we may have been able to clarify how kawaii-ness affects neurotransmitters such as dopamine and oxytocin, and brain activity (amygdala, prefrontal cortex, etc.) involved in emotion regulation and social bonding. Finally, our method was a cross-sectional analysis and, therefore, does not indicate causal relationships between the variables. For example, AI and VR experiments could have been used to examine the effects of kawaii-ness in a more controlled digital environment, potentially yielding more accurate data. Future research should incorporate longitudinal analysis methods to clarify the influence of brain state and kawaii-ness on happiness using information from a wider range of people of different ages, cities, and countries, as well as a variety of psychological scales, brain measurements, and intervention methods.

## 6. Conclusions

Path analysis was performed using brain healthcare quotients, FA-BHQ, GM-BHQ, and psychological data obtained from 182 healthy men and women. The results showed that kawaii-ness mediates the association between demographic variables and happiness. Furthermore, the correlation between happiness and the FA-BHQ centered on the limbic-thalamo-cortical pathway, which is responsible for emotional regulation, was confirmed. These results indicate the following: men, older people, and people engaged in knowledge work, and people with high annual incomes avoid kawaii-ness; as a result, they are unable to obtain the sense of happiness that they should have; as a result, they are unable to keep their brains healthy, and their brain functions, including emotional regulation, are not functioning properly; this may prevent them from maintaining or improving their performance.

## Figures and Tables

**Table 1:** Statistical differences between institutes.

	Group 1		Group 2			
	Mean	SD	Mean	SD	t	*p*
KRI	4.997	0.932	3.442	1.104	10.259	***
SHS	5.222	1.056	5.196	1.057	0.170	
Age	39.644	10.464	45.033	9.965	3.558	***
BMI	23.455	4.680	24.087	3.702	1.012	
Income	5.767	2.781	10.870	3.774	10.366	***
FA-BHQ	99.318	3.193	99.822	3.454	1.022	
GM-BHQ	105.210	8.508	100.767	8.146	3.599	***
	N	%	N	%	χ^2^	
Male	24	26.70%	62	67.40%	30.272	***
Female	66	73.30%	30	32.60%		
Knowledge work	26	28.90%	68	73.90%	36.930	***
Non-Knowledge work	64	71.10%	24	26.10%		

N = 182; *** *p* < 0.001. BMI: body mass index (BMI, kg/m^2^).

**Table 2:** Descriptive statistics and correlations.

	Variable	Mean	SD	1	2	3	4	5	6	7	8
1	KRI	4.211	1.284		0.251 **	−0.058	−0.051	0.044			
2	SHS	5.209	1.054	0.184 *		0.228 **	0.024	−0.033			
3	FA-BHQ	99.572	3.328	−0.107	0.133		0.211	−0.068			
4	GM-BHQ	102.964	8.597	0.324 ***	−0.016	0.189 *		−0.300 ***			
5	BMI	23.774	4.214	−0.160 *	−0.059	−0.038	−0.399 ***				
6	Knowledge work	0.517	0.501	−0.411 ***	0.094	0.098	−0.264 ***	0.147 *			
7	Income	8.346	4.184	−0.497 ***	0.009	0.137	−0.192 **	0.182 *	0.372 ***		
8	Age	42.368	10.539	−0.252 **	0.127	−0.223 **	−0.752 ***	0.216 **	0.216 **	0.117	
9	Sex	0.473	0.501	−0.530 ***	−0.139	0.128	−0.485 ***	0.234 **	0.431 ***	0.391 ***	0.218 **

N = 182; * *p* < 0.05; ** *p* < 0.01; *** *p* < 0.001. BMI: body mass index (BMI, kg/m^2^). The figures below the diagonal are Spearman’s rho. The figures above the diagonal are Pearson’s correlation coefficients controlling for demographic variables (knowledge work, income, age, and sex).

**Table 3:** Path coefficient. N = 182; * *p* < 0.05; ** *p* < 0.01; *** *p* < 0.001; ☨ *p* = 0.051. Figures are standardized path coefficients (β). BMI: body mass index (BMI, kg/m^2^).

Path			Path Coefficient
Sex	⇒	KRI	−0.322 ***
Income	⇒	KRI	−0.308 ***
Knowledge work	⇒	KRI	−0.129 ☨
Age	⇒	KRI	−0.139 *
Age	⇒	GM-BHQ	−0.671 ***
Sex	⇒	GM-BHQ	−0.315 ***
KRI	⇒	SHS	0.318 ***
BMI	⇒	GM-BHQ	−0.187 ***
Knowledge work	⇒	SHS	0.173 *
Age	⇒	SHS	0.171 *
GM-BHQ	⇒	FA-BHQ	0.395 ***
Sex	⇒	FA-BHQ	0.307 ***
SHS	⇒	FA-BHQ	0.218 **
			Covariance
Sex	⇔	Age	0.180 *
Knowledge work	⇔	Age	0.174 *
Knowledge work	⇔	Sex	−0.424 ***
Income	⇔	Sex	−0.358 ***
Knowledge work	⇔	Income	0.316 ***

**Table 4:** Summary of mediation analysis.

Relationship	Direct				Indirect				Conclusion
	Effect	Confidence Interval		*p*-Value	Effect	Confidence Interval		*p*-Value	
		Lower	Upper			Lower	Upper		
		Bound	Bound			Bound	Bound		
Knowledge work -> KRI -> SHS	0.192	0.021	0.355	0.030 *	−0.040	−0.102	−0.004	0.031 *	Partial Mediation
Income -> KRI -> SHS	0.131	−0.030	0.301	0.109	−0.096	−0.177	−0.040	0.000 ***	Full Mediation
Sex -> KRI -> SHS	−0.157	−0.325	0.016	0.074	−0.100	−0.187	−0.041	0.000 ***	Full Mediation
Age -> KRI -> SHS	0.186	0.045	0.315	0.011 *	−0.043	−0.105	−0.008	0.012 *	Partial Mediation

Standardized path coefficient (β). N = 182; * *p* < 0.05; *** *p* < 0.001.

**Table 5:** Partial correlations. N = 182; * *p* < 0.05; ** *p* < 0.01. All the paths are significant at the 5% level for multiple comparisons using the Benjamani–Hochberg method. The figures are Pearson’s partial correlation controlled for sex and GM-BHQ.

	Mean	SD	SHS
FA-BHQ	99.572	3.328	0.229 **
Corpus callosum	100.027	4.783	0.214 **
Internal capsule	100.160	3.988	0.193 *
Corona radiata	100.742	4.954	0.251 **
Posterior thalamic radiation	99.625	5.627	0.204 **
Cingulum	99.291	3.966	0.203 **
Superior longitudinal fasciculus	100.363	4.648	0.169 *
Uncinate fasciculus	98.751	6.164	0.210 **

## Data Availability

The data presented in this study are available on request from the corresponding author due to the need to protect the privacy of participants.
